# DICER1: A Key Player in Rheumatoid Arthritis, at the Crossroads of Cellular Stress, Innate Immunity, and Chronic Inflammation in Aging

**DOI:** 10.3389/fimmu.2018.01647

**Published:** 2018-07-24

**Authors:** Aurore De Cauwer, Alexandre Mariotte, Jean Sibilia, Seiamak Bahram, Philippe Georgel

**Affiliations:** ^1^Université de Strasbourg, INSERM, ImmunoRhumatologie Moléculaire UMR_S 1109, Fédération de Médecine Translationnelle de Strasbourg, Faculté de Médecine, Strasbourg, France; ^2^Fédération Hospitalo-Universitaire, OMICARE, Centre de Recherche d’Immunologie et d’Hématologie, Strasbourg, France; ^3^Centre de Référence des Maladies Autoimmunes Rares, Hôpitaux Universitaires de Strasbourg, Strasbourg, France

**Keywords:** Dicer1, inflammation, rheumatoid arthritis, senescence, ageing

## Abstract

Loss-of-function or knockout mouse models have established a fundamental role for the RNAse III enzyme DICER1 in development and tissue morphogenesis and/or homeostasis. These functions are currently assumed to result mainly from the DICER1-dependent biogenesis of microRNAs which exhibit important gene expression regulatory properties. However, non-canonical DICER1 functions have recently emerged. These include interaction with the DNA damage response (DDR) pathway and the processing of cytotoxic non-coding RNAs, suggesting that DICER1 might also participate in the regulation of major cellular processes through miRNA-independent mechanisms. Recent findings indicated that reduced *Dicer1* expression, which correlates with worsened symptoms in mouse models of joint inflammation, is also noted in fibroblast-like synoviocytes (FLS) harvested from rheumatoid arthritis (RA) patients, as opposed to FLS cultured from biopsies of osteoarthritic patients. In addition, low DICER1 levels are associated with the establishment of cellular stress and its associated responses, such as cellular senescence. Senescent and/or stressed cells are associated with an inflammatory secretome (cytokines and chemokines), as well as with “find-me” and “eat-me” signals which will attract and activate the innate immune compartment (NK cells, macrophages, and neutrophils) to be eliminated. Failure of this immunosurveillance mechanism and improper restauration of homeostasis could lead to the establishment of a systemic and chronic inflammatory state. In this review, we suggest that reduced DICER1 expression contributes to a vicious cycle during which accumulating inflammation and premature senescence, combined to inadequate innate immunity responses, creates the appropriate conditions for the initiation and/or progression of autoimmune-autoinflammatory diseases, such as RA.

## Biological Roles of DICER1

### The Canonical Role of DICER1: MicroRNA (miRNA) Biogenesis

**Figure 1:**
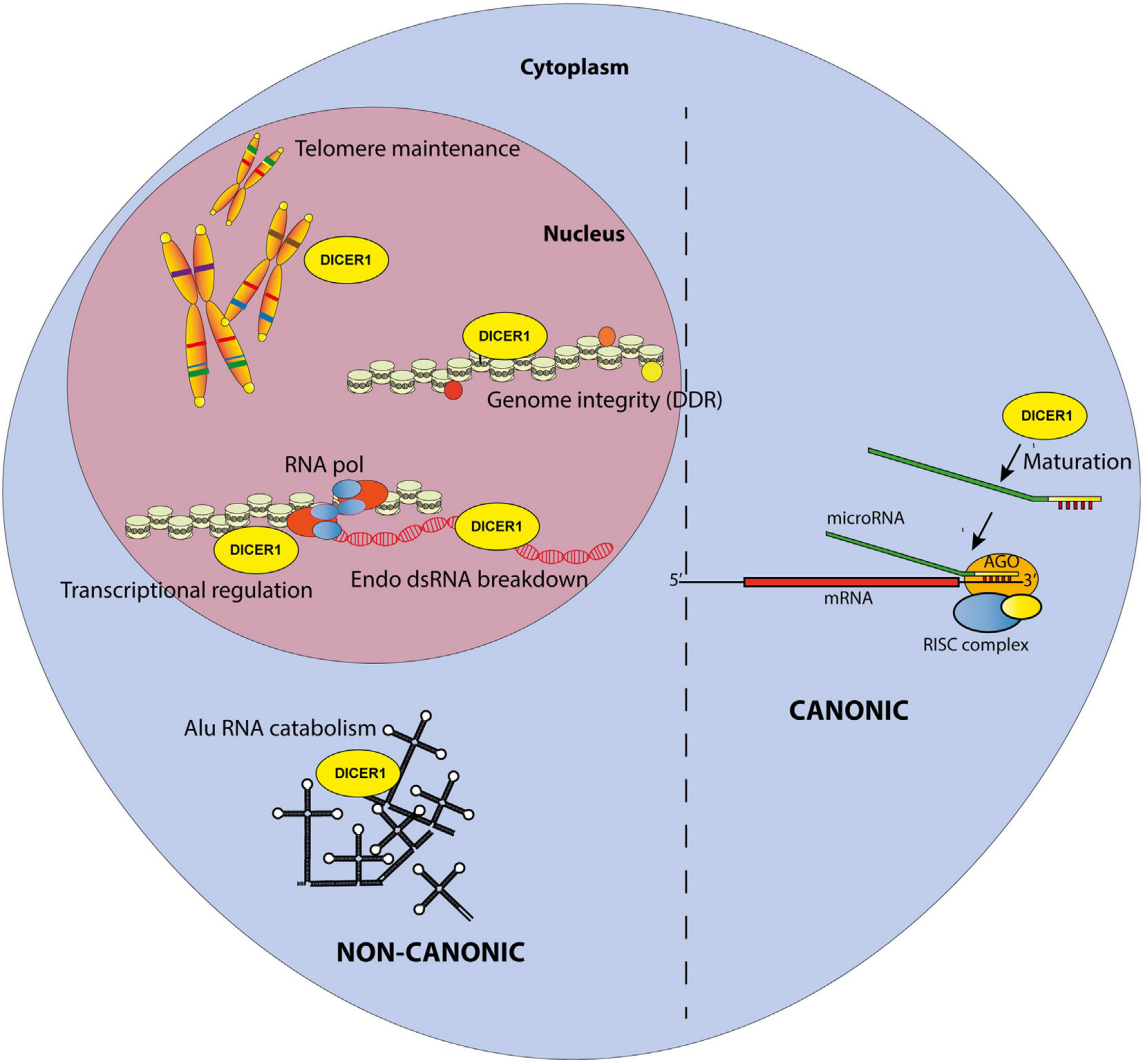
Canonical and non-canonical functions of DICER1 in the nucleus and the cytosol. The canonical function of DICER1 leads to microRNA maturation in the cytoplasm. Others functions are considered non-canonical.

Since its discovery by Bernstein et al. (1), the RNAse III enzyme DICER (encoded by the *DICER1* gene in *H. sapiens* and *Dicer1* in *Mus musculus*, the nomenclature that will be used throughout this review) has been extensively studied and its role in the miRNA biogenesis is today well described [reviewed in Ref. (2)]. miRNA synthesis usually begins with the RNA polymerase II-dependent transcription of genes encoding primary-miRNAs (pri-miRNAs), which are several kilobase-long stem-loop transcripts. Alternatively, pri-miRNAs can also originate from introns of protein coding genes. Whatever their origin, pri-miRNAs are then processed by the nuclear microprocessor complex DROSHA/DiGeorge syndrome Critical Region 8 (DGCR8) into precursor microRNAs (pre-miRNAs). Those 60–80 nucleotide-long precursors are then exported to the cytoplasm where they are recognized and cleaved by DICER1 into a 20–22 nucleotide-long RNA duplex (Figure 1). One miRNA strand is conserved and loaded into the RNA-induced silencing complex (RISC) composed of argonaute proteins. Guided by the miRNA, the RISC complex hybridizes with complementary mRNAs leading to either their degradation or translational inhibition. Therefore, DROSHA, DICER1, and miRNA are core factors of the Post-Transcriptional Gene Silencing process, a key regulatory mechanism of gene expression. In addition, several miRNAs are produced upon non-canonical pathway because their synthesis bypasses some of the aforementioned steps; those are Mirtons (whose synthesis is DROSHA-independent) (3) and miR-451, the only DICER1-independent miRNA described up to now (4, 5).

Interestingly, a study aiming at re-evaluating the contribution of the different key factors in miRNA biogenesis showed that while DROSHA is actually irreplaceable in the canonical miRNA synthesis, some miRNAs are still produced, albeit at reduced levels, without DICER1 (6). These observations, along with ours showing that reduced expression of DICER1 in fibroblast-like synoviocytes (FLS) from rheumatoid arthritis (RA) patients is associated with no more than a modest reduction of miRNA production (7), strongly suggest that other roles, besides miRNAs maturation, might be attributed to DICER1. Indeed, marked phenotypes have been observed in targeted (tissue-specific) *Dicer1* knockout mouse mutants, despite a noticeable preserved (and even sometimes increased) expression of many mature miRNAs.

### DICER1 Non-Canonical Roles

Accordingly, multiple reports have now described the existence of non-canonical, miRNA-independent, roles of DICER1 (Figure 1). Those functions are essentially implicated in nuclear RNAi and have been thoroughly reviewed elsewhere (8). In brief, DICER1, associated with TAR RNA Binding Protein and Protein activator of protein kinase R (PKR) (TRBP/PACT), was shown to regulate the transcription of a subset of hormone-inducible genes by interacting with their promoters in a dsRNA-dependent manner. Nuclear DICER1 is also implicated in the processing of endogenous dsRNA originating from overlapping transcription units, thereby protecting the cells from interferon (IFN)-mediated apoptosis. In addition, DICER1 plays an essential role in the maintenance of genome integrity (9), especially through interactions with the DNA damage response (DDR) pathway. It has been shown that in response to double-strand breaks in DNA, DICER1-dependent accumulation of break-specific dsRNAs facilitates the recruitment of reparation factors. Interestingly, this mechanism is also needed for the maintenance of telomeres (10).

Furthermore, the cytoplasm is also a major site of DICER1 non-canonical functions, which have been extensively studied over the last decade. A first hint for such roles was discovered in patients with age-related macular degeneration, which exhibit reduced DICER1 expression in retinal pigmented epithelium cells. In these cells, low *Dicer1* (but, importantly, not any of the other genes involved in miRNA production) expression triggered by shRNA knockdown in mice leads to cytotoxic accumulation of non-coding dsRNA formed upon the transcription Alu sequences (repetitive elements abundantly present in the human genome and classified as short interspersed nuclear elements (SINE)—retrotransposon family) (11). Accumulating Alu RNAs lead to a toll-like receptor (TLR)-independent, P2X7- and ROS-dependent activation of the NLRP3 inflammasome. The resulting maturation and secretion of IL-18 induces an MYD88-dependent pathway and caspase-8-mediated cell death, leading to macular degeneration (12–14).

Altogether, these data point to potentially devastating effects of *DICER1* mis-expression which can theoretically affect all steps of gene expression in both nuclear (replication/transcription/splicing) and cytoplasmic (translation) compartments.

## DICER1 in Inflammation

### miRNAs in Inflammation: Prominent Roles for miR-155 and -146a

There are 1,917 human miRNA sequences in the most recent miR database. This relatively large number, together with the capacity of every miRNA to target hundreds of mRNAs (15), indicates that they are able to virtually impact every biological function. It is therefore very much expected for miRNAs to be involved in most pathophysiological settings, among which inflammation and associated diseases were particularly scrutinized. In this context, miR-155 and -146a have been extensively described because they clearly exhibit crucial regulatory functions in innate and adaptive immunity. Indeed, miR-146a has been described as a mandatory regulator of the NF-κB pathway in T cells, targeting TRAF6 and IRAK1 (16). miR-146a was also correlated and functionally associated with the control of TNF-α production downstream of several TLRs and to the LPS tolerance phenomenon (17). In this report, it was notably observed that miR-146a is increased in human monocytic cells following LPS re-exposure. Until now, many groups found a pronounced inflammation-limiting role for miR-146a in various inflammatory settings, from atopic dermatitis (18) to sepsis (19). Strong evidence also attests that miR-146a participates in inflammatory disorders such as gout (20, 21) and RA [Ref. (22, 23) and see below].

miR-155, encoded by the *bic* locus, has been described as a major actor in inflammatory responses (24–26). miR-155 is considered a main driver of inflammatory responses through a large array of networks and its down-modulation is associated with termination of acute inflammation, as exemplified in the case of glucocorticoid treatments (27). Interestingly, the inflammatory effects of miR-155 are counteracted by miR-146a, as evidenced by a murine model where the deletion of the former is able to abrogate the inflammation induced by the loss of the latter (28). In essence, miR-155 and -146a, which roles were comprehensively analyzed in mouse knockout models, are considered as major players in the regulation of inflammatory responses.

Interestingly, miRNA biogenesis and the cellular stress response are tightly interconnected (29). This can be illustrated by the reciprocal interactions between type I IFNs-I, cytokines of paramount importance in the resolution of a virus-induced stress, which can modulate *DICER1* gene expression (30). In return, mice carrying a mutation in the *DICER1* gene exhibit an altered transcriptional profile of miRNA-regulated, IFN-stimulates genes (31). It is also noteworthy to observe that miR-124, a major player in the regulation of stress-induced genes in the brain (32), has recently been shown to modulate inflammation in a rat model of arthritis (33).

### Non-Canonical Roles of DICER1 in Inflammation

Evidence directly implicating non-canonical roles of DICER1 in inflammatory responses is scarce. To date, only two examples can be mentioned: (1) the DICER1-dependent processing of Alu RNAs which precludes the harmful activation of NLRP3 Inflammasome and the maturation/secretion of pro-inflammatory cytokines IL-1β and IL-18 (11) (see above) and (2) the involvement of nuclear DICER1 in the processing of dsRNA transcripts from overlapping loci, thus preventing an uncontrolled IFN response (34).

Regulation of IFN secretion and IFN-mediated responses are of high interest because excessive production of these cytokines is associated with several autoimmune diseases. Of note, dysregulation of *DICER1* expression has been linked to the modulation IFN responses, a feature which is considered to result from global miRNA deregulation. *DICER1* ablation in endometrial cancer cells was also linked to an increased IFN-β secretion and subsequent upregulation of IFN-stimulated genes (35). However, this response was interpreted as the consequence of cytoplasmic accumulation of pre-miRNAs which are able to trigger the activation of dsRNA sensors, hence leading to an IFN response. More recently, DICER1’s ablation in tumor-associated macrophages was shown to polarize the cells toward an M1-like phenotype associated with hyperactive IFNγ/STAT1 signaling. This observation, described as the result of decreased expression of the let-7 miRNA, can only be partially rescued by transduction with a lentivirus expressing let-7 (36). It is then conceivable that non-canonical roles of DICER1 might also play a role in the M1/M2 macrophage polarization. Nevertheless, this model, whereby unprocessed dsRNAs accumulate in the cytosol upon DICER1 deficiency and drive inflammatory responses, has been poorly explored so far.

Of note, an increase in cytoplasmic Alu RNA following stress promotes disassembly of stress granules (SGs) (37). Since SGs decrease the interactions between DICER1 and its co-factors, thereby reducing its activity (38), a cross-talk between stress-induced pathways and miRNA-independent functions of DICER1 appears also plausible. Furthermore, SGs negatively regulate the production of inflammatory cytokine such as IL-1β by controlling mRNAs stability and decay (39). Hence, impairment of this activity upon Alu RNA accumulation would also contribute to promote inflammation.

### DICER1 in Aging

Aging is an important risk factor for the development of inflammatory disorders/diseases (40). In rodents, aging has been associated with a decreased expression of *DICER1* in the adipose tissue (41). In human, octogenarians, compared with centenarians, exhibit global decrease in miRNA expression as well as reduced expression of miRNA biogenesis factors including DICER1 in blood cells (42, 43). However, these observations do not provide mechanistic insights for the contribution of DICER1 in the aging process. Of course, many miRNAs (such as miR-34) targeting emblematic pathways involved in senescence (e.g., P53/P21) have been described (44) and are likely to play a role in aging. Nevertheless, aging is a complex process characterized by nine hallmarks: genomic instability, telomere attrition, epigenetic alterations, loss of proteostasis, deregulated nutrient-sensing, mitochondrial dysfunction, cellular senescence, stem cell exhaustion, and altered intercellular communication (45), all of which are possibly impacted by *DICER1* misexpression, not only through impaired miRNAs maturation but also because non-canonical DICER1 functions may be affected as well.

With regards to genomic instability, the role of DICER1 in the processing of RNAs transcribed from retrotransposons belonging to long- or short-interspersed nuclear elements (line or SINE) families participates in the prevention of retrotransposition deleterious events (46). In addition, accumulation of Alu RNAs was found to restrain “stemcellness” and is associated with persistent DNA damage preventing tissue renewal (47). Their DICER1-dependent elimination is therefore required to maintain tissue homeostasis. Next, DICER1 is implicated in the DDR pathway by processing dsRNA essential for the DNA double-strand break repair. This process seems also to be necessary to prevent a second hallmark of aging, telomere shortening (10). Moreover, *DICER1* deletion has been associated with epigenetic alterations, such as chromatin remodeling, DNA methylation, and histone modification in mammalian cells (48, 49). Evidence in favor of a role of DICER1 in altered nutrient sensing and mitochondrial dysfunction is less documented. However, it was demonstrated that DICER1-depletion in adipocytes (i) overactivates the sensing signaling molecule mTORC1 and (ii) reduces mitochondria numbers, which are also irregularly shaped and associated with reduced oxidative metabolism in response to caloric restriction (50). As mentioned above, senescence has been amply described in relation to modified miRNA expression [e.g., Ref. (51)] but was also linked to Alu RNAs accumulation (47). Finally, downregulation of *Il-8* expression in endothelial cells upon *DICER1* knockdown (52) illustrates the potential impact of this multifunctional enzyme in the last hallmark of aging: cellular communication.

With regards to RA, normal aging of the immune system (immunosenescence) is associated with a higher risk to develop autoimmune disorders, including RA (53, 54). Alternatively, systemic joint inflammation may enhance the progression of immunosenescence and favor the development of comorbidities in RA patients (55).

## DICER1 and MIRNAS are Major Players in RA

Rheumatoid arthritis is a systemic autoimmune disease affecting around 1% of the global population. This rheumatic disease is characterized by multiple joint swelling, stiffness, and inflammatory pain, mainly in the small joints of hands and feet (56). Although the auto-immune feature of RA is clearly demonstrated, several decades of research have established a major role for the innate immune system and stromal cells in this disease (57). It is now commonly admitted that RA is a multifactorial disease, where its initiation and development requires concomitant participation of genetic, epigenetic, and environmental factors. Among epigenetic players involved in RA, miRNAs have been the focus of intense attention over the past decade (58).

There are presently more than 20 miRNAs, expression of which is deregulated in various cells (T cells, monocytes, and FLS)/compartments (blood and synovial fluid) harvested from RA patients (59, 60), and our lab has contributed to the identification of several of them within the miR-17~92 cluster (61–63). However, likely because RA etiology relies on innate and adaptive immune systems, miR-146a and -155, both of which have been involved in the regulation of adaptive (such as T cells-mediated) and inflammatory (e.g., in monocytes) responses, have been extensively studied in this disease. miR-146a is increased in RA patients (64–66) and is supposed to be integrated in a feedback loop, triggered by the unrestrained inflammation (67). Furthermore, murine models of RA have clearly shown that miR-146a restrains osteoclastogenesis (23). miR-155 is also upregulated in FLS and peripheral blood CD14-positive cells of RA patients (68, 69). In addition, its expression was correlated to the Disease Activity Score on 28 joints (DAS28) (70). Interestingly, miR-155 is also required for the development of the disease in the collagen-induced arthritis model, a commonly used mouse model of autoimmune arthritis (71). In FLS, upregulation of both miR-146a and miR-155 was correlated to negative regulation of osteoclastogenesis/MMP production. Therefore, this increased expression was interpreted as a way to limit the RA-associated osteoarticular destruction processes (64).

On the other hand, reduced miR-146a and -155 expression in regulatory T cells (Treg) has also been incriminated in RA (22), which illustrates that a global perturbation (driving either an overexpression or a down-modulation) of miRNA production is unlikely to represent a major trigger of RA pathogenesis. Indeed, increased (or decreased) miRs in activated T cells might be compensated by similar alterations in Tregs, and *vice versa*. In this regard, our observations indicating that (i) *Dicer1*-deficient mice exhibit worsened symptoms following experimental (upon K/BxN serum transfer) arthritis induction and (ii) that FLS cultivated from biopsies harvested in RA patients exhibit reduced *DICER1* expression (7) pinpoint to a potential involvement of non-canonical, miRNA-independent activities of DICER1 in joint inflammation. Several possibilities might be considered in line with the abovementioned roles of DICER1 in the processing of Alu sequences. For instance, abolishing DICER1 activity may lead to reduced production of Alu repeat-induced small RNAs (riRNAs) in the nucleus, thereby limiting the proliferative capacities of stem cells (72) and impairing tissue renewal in the joint. Combined with increased DNA damage (73) which is accompanied with the initiation of senescence, reduced DICER1 non-canonical activities might drive the accumulation of aged FLS resistant to apoptotic stimuli (7) and exhibiting pro-inflammatory capabilities [through IL-6, an essential component of the senescence-associated secretory phenotype (SASP) (74)], a dangerous cocktail likely driving their aggressive phenotype observed in RA patients. As mentioned above, *Dicer1* expression is negatively regulated by inflammatory cytokines such as type I IFNs, further aggravating the inflammatory response.

## DICER1 at the Crossroads Between Senescence and Inflammation in RA

**Figure 2:**
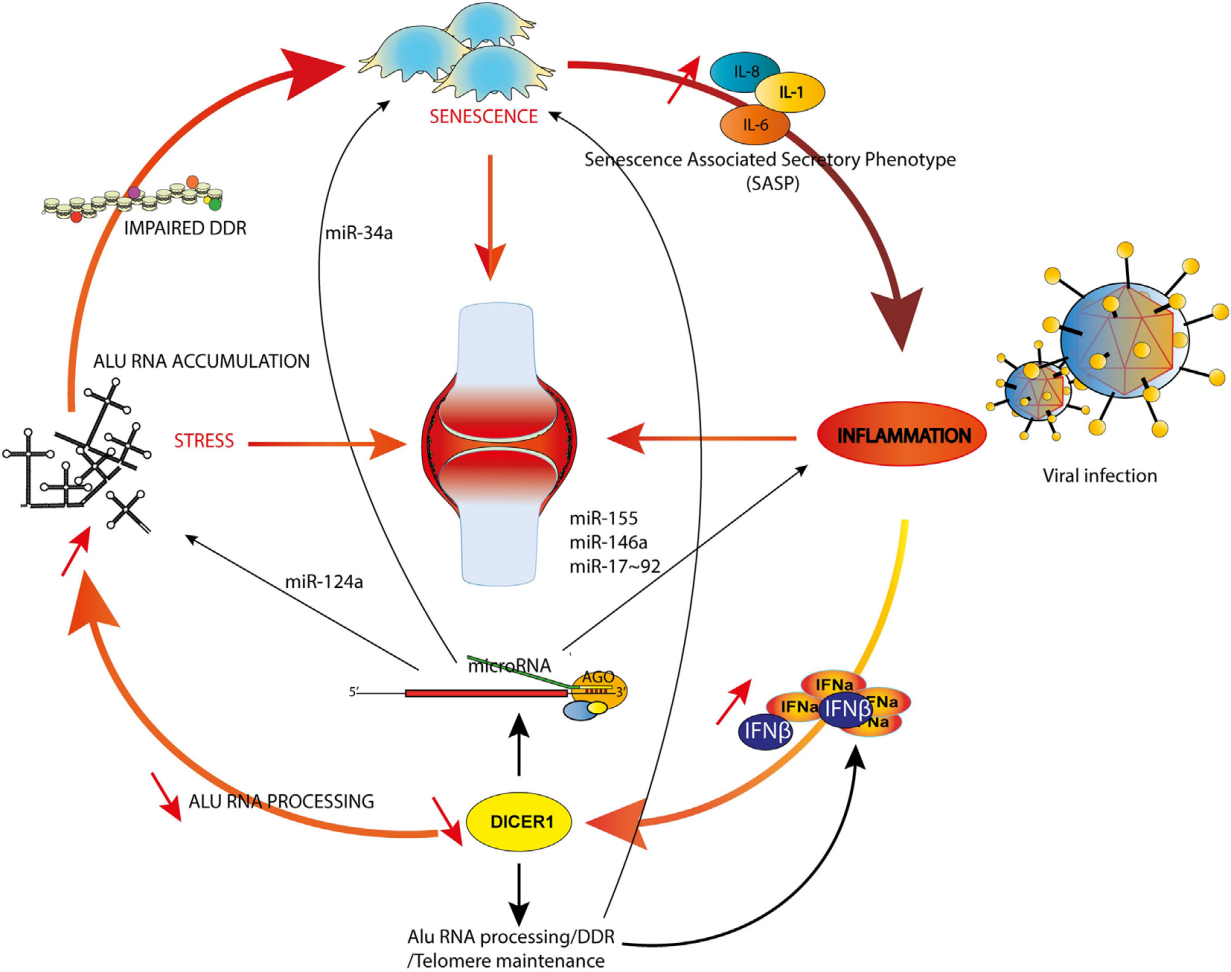
DICER1 functions at the crossroads of inflammation, senescence and aging. Examples of microRNAs involved in both rheumatoid arthritis (RA) and inflammation (miR-155 and -146a), RA and senescence (miR-34a) or RA and stress (miR-124a) are shown. The model illustrates how an initial trigger (e.g., a viral infection) might initiate a vicious circle (see text).

These multiple interactions are integrated in the model illustrated in Figure 2. We considered three main triggers (or hallmarks) of RA, aging (75), inflammation [through specific cytokines (76)], and stress (77) and their reciprocal interconnections mediated by canonical and non-canonical functions of DICER1. For sake of simplicity, we emphasized only specific miRNAs and other DICER1 ligands (such as Alu RNAs), but nevertheless, our model supports the notion that DICER1 alterations might perturb every trigger of RA and initiate a chain reaction actually driving pathogenesis. Indeed, their interactions create the appropriate conditions to build a vicious circle which can for instance, start with IFN-dependent *DICER1* down-modulation (for example, as a result of a chronic viral infection). This initial event might contribute to accumulation of Alu RNAs and other dsRNAs in the cytoplasm, which favor the survival of senescent cells in which DNA damages are poorly fixed (hence increasing the inflammatory settings through the SASP) and reduced formation of SGs, leading to an impaired degradation of viral RNAs. Viral RNAs and cytoplasmic endogenous dsRNAs will ultimately enhance type I IFN production and amplify the vicious circle. Of course, this model is incomplete and could also, for instance, integrate metabolism (or other environmental factors contributive to RA). Interestingly, interactions between PKR, TRBP, and DICER1 were described, suggesting an additional (miRNA-independent) role for DICER1 in immunometabolism (78).

## Concluding Remarks

Precisely evaluating the various roles of DICER1 appears a challenging task due to its complex and pleiotropic roles. Furthermore, factors that influence *DICER1* gene expression in specific cells and at defined developmental stages are still poorly described. In addition, *DICER1* transcripts and protein levels are not always correlated (79), adding another layer of complexity. Moreover, DICER1 activity appears regulated by post-translational modification such as phosphorylation and SUMOylation (80), and the protein can shuttle between the cytosol and the nucleus and exert different activities in these two compartments, depending on associations with various co-factors. For instance, DICER1-efficient processing activity of Alu RNAs depends on poly(C)-binding protein 2 binding, which is inhibited by iron overload (81).

Here, we provided several examples of reciprocal interactions between DICER1 and mechanisms (stress, inflammation, and aging) that can be either considered as triggers (or inducers of *DICER1* expression) or effectors (i.e., that are able to respond to DICER1-dependent products such as miRNAs or metabolites of Alu or other long non-coding RNAs). We suggest that within this complex network of interactions, DICER1 occupies a central position. In this model, perturbations of these interactions modify homeostasis and drive pathogenesis. The focus of this review has been RA, but this network can be extended to other age-dependent pathological conditions, beyond autoimmune or inflammatory diseases, such as cancer or neurodegeneration.

## Author Contributions

PG, AC, AM, JS, and SB participated in discussions; drafted and approved the manuscript.

## Conflict of Interest Statement

The authors declare that the research was conducted in the absence of any commercial or financial relationships that could be construed as a potential conflict of interest.
